# Urinary cell cycle arrest biomarkers and chitinase 3-like protein 1 (CHI3L1) to detect acute kidney injury in the critically ill: a post hoc laboratory analysis on the FINNAKI cohort

**DOI:** 10.1186/s13054-020-02867-w

**Published:** 2020-04-10

**Authors:** Eric A. Hoste, Suvi T. Vaara, Jorien De Loor, Mikko Haapio, Lieve Nuytinck, Kristel Demeyere, Ville Pettilä, Evelyne Meyer, The FINNAKI Study Group, Raili Laru-Sompa, Anni Pulkkinen, Minna Saarelainen, Mikko Reilama, Sinikka Tolmunen, Ulla Rantalainen, Marja Miettinen, Markku Suvela, Katrine Pesola, Pekka Saastamoinen, Sirpa Kauppinen, Ville Pettilä, Kirsi-Maija Kaukonen, Anna-Maija Korhonen, Sara Nisula, Suvi Vaara, Raili Suojaranta-Ylinen, Leena Mildh, Mikko Haapio, Laura Nurminen, Sari Sutinen, Leena Pettilä, Helinä Laitinen, Heidi Syrjä, Kirsi Henttonen, Elina Lappi, Hillevi Boman, Tero Varpula, Päivi Porkka, Mirka Sivula, Mira Rahkonen, Anne Tsurkka, Taina Nieminen, Niina Pirttinen, Ari Alaspää, Ville Salanto, Hanna Juntunen, Teija Sanisalo, Ilkka Parviainen, Ari Uusaro, Esko Ruokonen, Stepani Bendel, Niina Rissanen, Maarit Lång, Sari Rahikainen, Saija Rissanen, Merja Ahonen, Elina Halonen, Eija Vaskelainen, Meri Pouk-kanen, Esa Lintula, Sirpa Suominen, Jorma Heikki-nen, Timo Lavander, Kirsi Heinonen, Anne-Mari Juopperi, Tadeusz Kaminski, Fiia Gäddnäs, Tuija Kuusela, Jane Roiko, Sari Karlsson, Matti Reinikainen, Tero Surakka, Helena Jyrkönen, Tanja Eiserbeck, Jaana Kallinen, Tero Ala-Kokko, Jouko Laurila, Sinikka Sälkiö, Vesa Lund, Päivi Tuominen, Pauliina Perkola, Riikka Tuominen, Marika Hietaranta, Satu Johansson, Seppo Hovilehto, Anne Kirsi, Pekka Tiainen, Tuija Myllärinen, Pirjo Leino, Anne Toropainen, Anne Kuitunen, Jyrki Tenhunen, Ilona Leppänen, Markus Levoranta, Sanna Hoppu, Jukka Sauranen, Atte Kukkurainen, Samuli Kortelainen, Simo Varila, Outi Inkinen, Niina Koivuviita, Jutta Kotamäki, Anu Laine, Simo-Pekka Koivisto, Raku Hautamäki, Maria Skinnar

**Affiliations:** 1 Intensive Care Unit, Ghent University Hospital, 2K12, Route 1280a, C. Heymanslaan 10, 9000 Ghent, Belgium; 2 Research Fund-Flanders (FWO), Egmontstraat 5, 1000 Brussel, Belgium; 3 Division of Intensive Care Medicine, Department of Anaesthesiology, Intensive Care and Pain Medicine, University of Helsinki and Helsinki University Hospital, Box 340, 00029 Helsinki, Finland; 4 Division of Nephrology, Abdominal Center, University of Helsinki and Helsinki University Hospital, Box 340, FI-00029 HUS Helsinki, Finland; 5 Faculty of Medicine and Health Sciences, Health Innovation and Research Institute of the Ghent University Hospital (UZ Gent) (HIRUZ) Ghent University Hospital, C. Heymanslaan 10, 9000 Ghent, Belgium; 6 Department of Pharmacology, Toxicology and Biochemistry, Laboratory of Biochemistry, Faculty of Veterinary Medicine, Ghent University, Salisburylaan 133, 9820 Merelbeke, Belgium

**Keywords:** Acute kidney injury, Biological markers, Biomarkers, Chitinase, Lipocalins, Intensive care, NephroCheck

## Abstract

**Background:**

Acute kidney injury (AKI) is a frequently occurring syndrome in critically ill patients and is associated with worse outcomes. Biomarkers allow early identification and therapy of AKI which may improve outcomes. Urine chitinase 3-like protein 1 (uCHI3L1) was recently identified as a promising urinary biomarker for AKI. In this multicenter study, we evaluated the diagnostic performance for AKI stage 2 or greater of uCHI3L1 in comparison with the urinary cell cycle arrest biomarkers urinary tissue inhibitor of metalloproteinases-2 (TIMP-2)•insulin-like growth factor-binding protein 7 (IGFBP7) measured by NephroCheck Risk®.

**Methods:**

Post hoc laboratory study of the prospective observational FINNAKI study. Of this cohort, we included patients with stored admission urine samples and availability of serum creatinine at day 1 of admission. Patients who already had AKI stage 2 or 3 at ICU admission were excluded. AKI was defined and staged according to the KDIGO definition and staging system. The primary endpoint was AKI stage 2 or 3 at day 1. Biomarker performance was assessed by the area under the curve of the receiver operating characteristic curve (AUC). We assessed individual performance and different combinations of urine biomarkers.

**Results:**

Of 660 included patients, 49 (7.4%) had AKI stages 2–3 at day 1. All urine biomarkers were increased at admission in AKI patients. All biomarkers and most combinations had AUCs < 0.700. The combination uCHI3L1•TIMP-2 was best with a fair AUC of 0.706 (0.670, 0.718). uCHI3L1 had a positive likelihood ratio (LR) of 2.25 which was comparable to that of the NephroCheck Risk® cutoff of 2.0, while the negative LR of 0.53 was comparable to that of the NephroCheck Risk® cutoff of 0.3.

**Conclusions:**

We found that uCHI3L1 and NephroCheck Risk® had a comparable diagnostic performance for diagnosis of AKI stage 2 or greater within a 24-h period in this multicenter FINNAKI cohort. In contrast to initial discovery and validation studies, the diagnostic performance was poor. Possible explanations for this observation are differences in patient populations, proportion of emergency admissions, proportion of functional AKI, rate of developing AKI, and observation periods for diagnosis of AKI.

## Background

Acute kidney injury (AKI) is a commonly occurring syndrome in critically ill patients admitted to the intensive care unit (ICU) and is associated with increased morbidity and mortality [1]. Recent studies indicate that the incidence of AKI in ICU patients varies between 39 and 56% when defined by the Kidney Disease: Improving Global Outcomes (KDIGO) consensus criteria [2–4]. These criteria define AKI based upon an increase of plasma creatinine concentration (Cr) or a period of decreased urine output (UO), and so reflect changes in glomerular filtration rate (GFR). Kidney biomarkers indicate stress or damage to the kidney tubular cells, thus giving additional information potentially at an earlier stage than Cr or UO [5]. This may be of importance since more early identification and stratification of therapy by the use of AKI biomarkers can improve outcomes [6, 7].

At present, the 2-biomarker panel of the cell cycle arrest biomarkers urinary tissue inhibitor of metalloproteinases-2 (TIMP-2)•insulin-like growth factor-binding protein 7 (IGFBP7) has shown the best predictive value for AKI in general ICU patients [5]. Given, the heterogeneous etiology of AKI and also different patients’ baseline characteristics, different biomarkers may be complimentary to each other. Recently, we showed in a single-center prospective cohort study that urinary chitinase 3-like protein 1 (uCHI3L1) is a promising AKI biomarker in ICU patients when compared to urinary neutrophil gelatinase-associated lipocalin (uNGAL) [8]. A subsequent study by our group in adult patients who underwent elective cardiac surgery demonstrated that in this specific setting, both uCHI3L1 and uNGAL showed inadequate predictive value for AKI [9].

The aim of this study was to evaluate uCHI3L1 as a biomarker for AKI in ICU patients included in the multicenter Finnish Acute Kidney Injury (FINNAKI) study and compare this to the NephroCheck Risk® score, the composite of the cell cycle arrest biomarkers TIMP-2 and IGFBP7. This may provide external validation to the biomarker uCHI3L1 that showed promise in our single-center study [8].

## Methods

This was a post hoc laboratory study of the prospective, observational FINNAKI study conducted in 17 Finnish intensive care units (ICUs) between September 1, 2011, and February 1, 2012 [4]. The Ethics Committee of the Department of Surgery at the Helsinki University Hospital gave a nationwide approval for the study. To include patients, we used deferred consent and as soon as possible obtained a written, informed consent. Each patient or his/her next of kin gave a written, informed consent. The study was conducted according to the Declaration of Helsinki. Reporting is according to the STROBE guidelines (suppl. data) [10].

## Patients

**Fig. 1:**
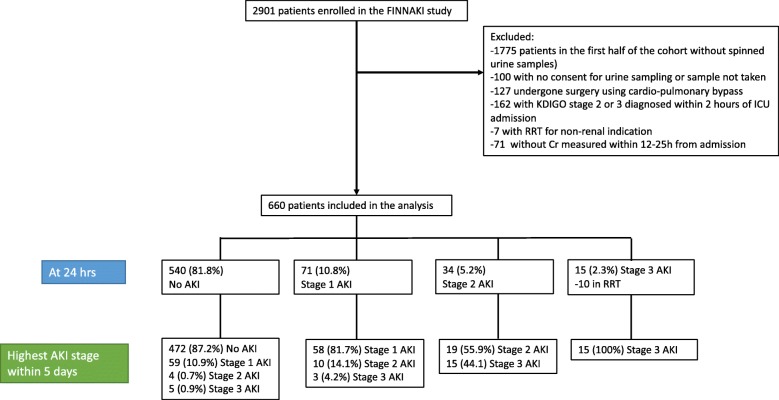
Patients’ flow chart

We enrolled all patients with an emergency admission to the ICU of any duration or an elective post-surgical admission expected to last over 24 h in the primary study [4]. Patients were excluded if they (1) had end-stage renal disease requiring maintenance dialysis; (2) were organ donors; (3) received intermediate care, since our focus was on critically ill patients; (4) had received renal replacement therapy (RRT) while enrolled in the study during previous ICU admission; (5) were transferred from another ICU where the data collection for the study was fulfilled; or (6) were not permanently living in Finland or were unable to give consent due to insufficient language skills as informed consent needs to be given using mother tongue. From the current analysis, we further excluded patients who (1) were enrolled to the study before December 1, 2011, and did not have centrifuged urine samples; (2) did not consent for urine sampling or where no sample was taken; (3) had undergone surgery with cardiopulmonary bypass; these were excluded as uCHI3L1 proved of less value in this cohort in a previous study; (4) had already stage 2 or 3 AKI within 2 h of ICU admission; (5) were treated with RRT for non-renal indications, as this would lead to false positives when non-renal use of RRT is classified as AKI; or (6) did not have a blood sample taken at 24 h from admission and also no routine Cr measurement taken 12 to 25 h from admission. If one of these samples was present, the patient was included (Fig. 1).

### Sample collection

Urine samples were collected within 2 h once the patient was admitted to the ICU. Additionally, blood and urine were collected 24 h later if the patient was still in the ICU. Urine samples were centrifuged and frozen in aliquots. Kidney biomarkers were analyzed on urine samples that were collected within 2 h. Blood samples were collected in EDTA tubes and centrifuged, aliquoted, and frozen. Samples were kept in − 80° until analyzed.

### Laboratory assays

The uCHI3L1, urinary TIMP-2, urinary IGFBP7, and NephroCheck Risk® analyses were performed in 2018. The laboratory analyses were performed blinded to the clinical data and the KDIGO classification. We measured the concentration of uCHI3L1 by a sandwich enzyme-linked immunosorbent assay (ELISA) technique (DC3L10, R&D Systems, Minneapolis, MN, USA). With the Astute140® Meter, we measured the concentrations of urinary TIMP-2, urinary IGFBP7, and NephroCheck Risk® by a fluorescent immunoassay technique (NephroCheck® Test, Astute Medical, San Diego, CA, USA). Details on these laboratory analyses were recently described [9].

In addition, we also evaluated the combinations of uCHI3L1 and the individual cell cycle arrest biomarkers and the NephroCheck Risk® for the prediction of AKI stage 2 or greater. Similar to the NephroCheck Risk®, this was done by multiplying the concentrations of individual biomarkers, e.g., uCHI3L1 conc x TIMP-2 concentration.

### Data collection

We collected data on patients’ baseline characteristics and risk factors for AKI with case report forms. These data were supplemented by physiological and laboratory data, disease severity scores, and ICU diagnoses from the ICU data management system via the Finnish Intensive Care Consortium database. The attending clinicians screened the patients for the presence of sepsis according to the ACCP/SCCM definition until day 5 [11]. The presence of pre-existing chronic kidney disease (CKD) was defined as GFR < 60 mL/min/1.73m^2^ as mentioned in patients’ medical records.

### Diagnosis of AKI and study endpoints

In the FINNAKI study, we classified AKI according to the KDIGO definition on routinely measured Cr and hourly UO as well as RRT in the ICU. As baseline Cr, we used the most recent value obtained within a year but at least a week preceding ICU admission. If baseline Cr was not available and patients had no previous history of CKD, a Modification of Diet in Renal Disease (MDRD) equation-derived baseline creatinine was used, assuming a minimum GFR of 75 mL/min/1.73m^2^ following the Acute Dialysis Quality Initiative (ADQI) and KDIGO recommendations [2, 12]. The study protocol did not include scheduled Cr measurements, and all data were observational. Cr was generally measured once daily in the morning lab tests. For the purpose of the current analysis, we analyzed all available plasma samples taken at 24 h for Cr (*n* = 588 patients). Additionally, 72 patients had Cr measured as part of the ICU lab routines within 12 to 25 h from ICU admission. Thus, an index Cr measured at least 12 h but not more than 25 h from admission was available from 660 patients. All these patients also had urine output recordings at least for 12 h and data about RRT use in the ICU. Based on these data, we formed the primary endpoint of the analysis: AKI stage 2 or 3 at 24 h.

As a sensitivity analysis, we analyzed the biomarker performance to predict (1) AKI of any severity at 24 h, (2) AKI of any severity at 48 h, and (3) stage 2 or 3 AKI at 48 h. The latest Cr values and UO recordings available up to 48 h from ICU admission were used to define the patient’s AKI status at 48 h. Longer time periods were not considered, since multiple events may contribute to the occurrence of AKI. If these occur after the measurement of the biomarker, there will be a negative biomarker reading. These patients will so incorrectly be classified as false negatives: negative biomarker and still occurrence of AKI.

### Statistical analysis

We report the non-normally distributed continuous data as median with interquartile range (IQR) and categorical data with count and percentage. We compared continuous data using Mann-Whitney *U* test and categorical data with Fisher’s exact test or chi-square test where appropriate. We assessed the predictive ability of the biomarkers and combinations for the primary endpoint by calculating the area under the receiver-operator characteristic curve (AUC-ROC) and compared these according to the method of DeLong et al. and with the calculation of binomial exact 95% confidence intervals [13]. For uCHI3L1 and the combination of this biomarker with the other biomarkers, we assessed a cutoff based on the Youden index [14]. We considered a two-sided *p* value less than 0.05 as significant and did not correct for multiple comparisons.

We pre-defined the AUC as follows: excellent, 0.900 ≤ AUC-ROC ≤ 1; good, 0.800 ≤ AUC-ROC ≤ 0.899; fair, 0.700 ≤ AUC-ROC ≤ 0.799; poor, 0.600 ≤ AUC-ROC ≤ 0.699; and failed when AUC-ROC < 0.600 [15].

Analyses were conducted using SPSS Statistics 23.0 for Microsoft and 24.0 for Mac (IBM, Armonk, NY), MedCalc Statistical Software version 19.0.4 (MedCalc Software bvba, Ostend, Belgium), and RStudio 1.1.456 for Mac.

## Results

A total of 660 patients were included in this study (Fig. 1). Of these, 120 (18.2%) patients had AKI at time 24 h after ICU admission. Their baseline characteristics are provided in Table 1. Of these patients, 96% were emergency admissions, 70% received mechanical ventilation, and 54.2% of them were administered norepinephrine. Altogether, 21 patients (3.2%) had AKI stage 1 diagnosed within 2 h of ICU admission. Of these, 5 patients had AKI stage 2 or 3 at 24 h and 4 at 48 h.

**Table 1:** Baseline characteristics of patients

	Data available	All patients	AKI stage 2 or 3 at 24 h (*n* = 49)	AKI stage 0 or 1 at 24 h (*n* = 611)	*p* value
Age (years)	660	64 [53–73]	68 [57–77]	64 [52–73]	0.038
Male sex	660	410/660 (62.1%)	35/49 (71.4%)	375/611 (61.4%)	0.172
BMI (kg/m^2^)	658	26.1 [23.4–29.4]	26.3 [24.4–30.9]	26.1 [23.4–29.4]	0.275
Baseline creatinine (μmol/L)	393	75 [61–95]	81 [68–107]	75 [60–93]	0.028
Baseline Cr with missing values imputed* (μmol/L)	660	82 [69–95]	90 [74–99]	81 [69–95]	0.058
Diabetes mellitus	660	139/660 (21.2%)	11/49 (22.4%)	128/611 (20.9%)	0.855
Arteriosclerosis	650	90/650 (13.6%)	10/48 (20.8%)	80/602 (13.3%)	0.189
CKD	659	41/659 (6.2%)	4/49 (8.2%)	37/610 (6.1%)	0.535
Chronic liver disease	654	20/654 (3.0%)	1/49 (2.0%)	19/605 (3.1%)	> 0.999
Hypertension	652	304/652 (46.1%)	24/49 (49.0%)	280/603 (46.4%)	0.767
Systolic heart failure	651	61/651 (9.2%)	6/48 (12.5%)	55/603 (9.1%)	0.438
Chronic obstructive pulmonary disease	656	73/656 (11.1%)	7/49 (14.3%)	66/607 (10.9%)	0.477
Pre-ICU AKI risk factors**					
Sepsis	660	144/660 (21.8%)	14/49 (28.6%)	130/611 (21.3%)	0.279
Hypovolemia	657	179/657 (27.1%)	23/49 (46.9%)	156/608 (25.7%)	0.002
Cardiopulmonary resuscitation	660	86/660 (13.0%)	6/49 (12.2%)	80/611 (13.1%)	> 0.999
Massive transfusion	660	16/660 (2.4%)	4/49 (8.2%)	12/611 (2.0%)	0.025
Acute liver failure	660	10/660 (1.5%)	3/49 (6.1%)	7/611 (1.1%)	0.032
Cardiogenic shock	660	19/660 (2.9%)	7/49 (14.3%)	12/611 (2.0%)	< 0.001
Radiocontrast	657	189/657 (28.6%)	14/48 (29.2%)	175/609 (28.7%)	> 0.999
Diuretics	639	190/639 (28.8%)	17/47 (36.25%)	173/592 (29.2%)	0.323
Angiotensin converting enzyme inhibitor or angiotensin receptor blocker	645	145/645 (22.5%)	13/48 (27.1%)	132/597 (22.1%)	0.472
Non-steroidal anti-inflammatory drug	622	61/622 (9.8%)	5/45 (11.1%)	56/577 (9.7%)	0.793
Hydroxyethyl starch	645	78/645 (12.1%)	17/49 (34.7%)	61/596 (10.2%)	< 0.001
Peptidoglycan antibiotic	659	28/659 (4.2%)	5/49 (10.2%)	23/610 (3.8%)	0.049
Emergency admission	660	635/660 (96.2%)	44/49 (89.8%)	591/611 (96.7%)	0.031
Surgical admission	660	191/660 (29.1%)	22/49 (44.9%)	170/611 (27.8%)	0.014
Admitted from					
Operation room or recovery	660	177/660 (26.8%)	22/49 (44.9%)	155/611 (25.4%)	0.004
Emergency department	660	272/660 (41.2%)	8/49 (16.3%)	264/611 (43.2%)	< 0.001
Ward	660	131/660 (19.8%)	15/49 (30.6%)	116/611 (19.0%)	0.062
Other ICU/high-dependency unit/others	660	80/660 (12.1%)	4/49 (8.2%)	76/611 (12.4%)	0.497
SAPS II (24 h)	660	38 [29–49]	52 [37–63]	37 [28–48]	< 0.001
SOFA (24 h)	660	7 [4–9]	10 [8–13]	6 [4–9]	< 0.001
Mechanical ventilation during ICU stay	660	462/660 (70.0%)	43/49 (87.8%)	419/611 (68.6%)	0.003
Norepinephrine (within 24 h)	660	358/660 (54.2%)	43/49 (87.8%)	315/611 (51.6%)	< 0.001

*MDRD equation assuming GRF ≥ 75 mL/min/1.73 m^2^. Imputed for 16 patients with endpoint+ and 251 with endpoint−

**Pre-ICU AKI risk factors if present in 48 h preceding ICU admission

KDIGO AKI stage 2–3 criteria were met by 49 patients (7.4%) at 24 h (i.e., the primary endpoint). Of those 49, AKI criteria were met by 27 (55.1%) using Cr criteria and in 29 (59.1%) using UO criteria; 10 patients (20.4%) were treated with RRT within the first 24 h after ICU admission. AKI was diagnosed by Cr criteria only in 15 patients (30.6%) and UO criteria only in 17 patients (34.7%), 12 patients (24.5%) met both Cr and UO criteria, and in 5 patients, AKI was diagnosed on use of RRT only.

Patients with AKI stage 2 or greater were older and had higher baseline creatinine compared to patients with AKI stages 0–1. AKI stage 2–3 patients had more risk factors for AKI such as hypovolemia, massive transfusion, acute liver failure, cardiogenic shock, administration of hydroxyethyl starch or peptidoglycan antibiotics, and emergency or surgical admission. Finally, AKI stage 2–3 patients had higher SAPS II and SOFA scores and received more often mechanical ventilation and/or norepinephrine.

### Urinary biomarkers for early diagnosis of AKI stage 2 or 3 at 24 h

Patients with AKI stage 2 or greater at 24 h had higher concentrations for all urinary biomarkers and for the different combinations thereof measured at ICU admission (Table 2).

**Table 2:** Biomarker levels. Median [IQR] biomarker concentrations in patients according to positive or negative primary endpoint (KDIGO stage 2 or 3 AKI at 24 h)

	AKI stage 2 or 3 at 24 h (*n* = 49)	AKI stage 0 or 1 at 24 h (*n* = 611)	*p* value
uCHI3L1 (ng/mL)	3.92 [0.12–12.45]	0.44 [0.12–2.42]	< 0.001
TIMP-2 (ng/mL)	6.40 [3.15–10.15]	3.40 [1.80–6.10]	< 0.001
IGFBP7 (ng/mL)	109.0 [46.9–300.9]	64.3 [32.4–128.9]	0.01
NephroCheck Risk® (ng/mL)^2^/1000	0.96 [0.19–2.64]	0.22 [0.06–0.85]	< 0.001
NephroCheck Risk® > 0.3 and ≤ 2.0 (ng/mL)^2^/1000	22/49 (44.9%)	190/611 (31.1%)	< 0.001
NephroCheck Risk® > 2.0 (ng/mL)^2^/1000	14/49 (28.6%)	75/611 (12.3%)	
uCHI3L1•TIMP-2 (ng/mL)^2^	19.18 [1.34–113.50]	1.24 [0.27–13.95]	< 0.001
uCHI3L1•IGFB7 (ng/mL)^2^	199.6 [19.4–2932.9]	20.9 [5.1–221.7]	< 0.001
uCHI3L1•NephroCheck Risk® (ng/mL)^3^/1000	2.04 [0.11–28.13]	0.09 [0.01–1.31]	< 0.001

**Fig. 2:**
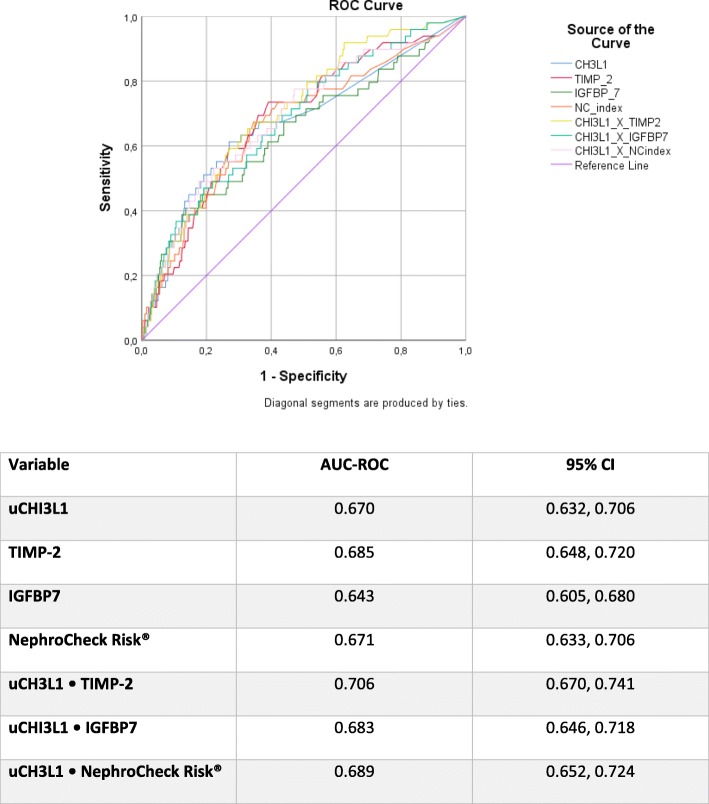
ROC curves for biomarkers for the primary endpoint: AKI stage 2 or 3 at 24 h (*n* = 49 positive)

The AUC-ROCs for AKI stage 2 or greater at 24 h were poor (< 0.700) for all biomarkers and their combinations, except for the combination uCHI3L1•TIMP-2 with a fair AUC-ROC of 0.706 (Fig. 2). This AUC-ROC was not statistically significantly higher than those of either uCHI3L1 or NephroCheck Risk® alone.

Based on a Youden analysis, the best cutoff for uCHI3L1 was assessed, and these sensitivity and specificity values were found to be in between those of the NephroCheck Risk® cutoff values of 0.3 and 2.0 (Table 3) [16]. The positive likelihood ratio (LR) of AKI stage 2 or greater for uCHI3L1 was comparable to that of the NephroCheck Risk® 2.0 cutoff, while the negative LR was comparable to that of the NephroCheck Risk® 0.3 cutoff (Table 3). Combining uCHI3L1 either with IGFBP7 or with the NephroCheck Risk® resulted in an increase of the specificity, a decrease of sensitivity, and the highest positive LRs.

**Table 3:** Diagnostic performance of biomarkers and biomarker combinations for AKI defined by KDIGO occurring at 24 h

Biomarker	Cutoff value	Sensitivity	Specificity	Positive LR	Negative LR
uCHI3L1 (ng/mL)	> 2.1	61.2% (46.2, 74.8)	72.8% (69.1, 76.3)	2.25 (1.74, 2.92)	0.53 (0.37, 0.76)
NephroCheck Risk® (ng/mL)^2^/1000	> 0.3	73.5% (58.9, 85.1)	56.6% (52.6, 60.6)	1.69 (1.34, 2.05)	0.47 (0.29, 0.75)
NephroCheck Risk® (ng/mL)^2^/1000	> 2.0	28.6% (16.6, 43.3)	87.7% (84.9, 90.2)	2.33 (1.43, 3.80)	0.81 (0.68, 0.97)
uCHI3L1•TIMP-2 (ng/mL)^2^	> 5.5	65.3% (50.4, 78.3)	67.3% (63.4, 71.0)	2.00 (1.58, 2.52)	0.52 (0.35, 0.76)
uCHI3L1•IGFBP7 (ng/mL)^2^	> 459.8	46.9% (32.5, 61.7)	80.7% (77.3, 83.7)	2.43 (1.73, 3.41)	0.66 (0.50, 0.86)
uCH3L1• NephroCheck Risk® (ng/mL)^3^/1000	> 3.3	48.9% (34.4, 63.7)	82.2% (78.9, 85.1)	2.75 (1.97, 3.83)	0.62 (0.47, 0.82)

### Sensitivity analyses

The AUC-ROCs for AKI of any severity of AKI at 24 h for all biomarkers and their combinations were poor (< 0.700). Sensitivity, specificity, and positive and negative likelihood ratios were corresponding to that of the primary endpoint (Additional file table 1). The results regarding AKI of any severity at 48 h (Additional file Table 2) and stage 2 or 3 AKI at 48 h (Additional file table 3) corroborated that of the primary endpoint.

## Discussion

In this multicenter validation study, we found that uCHI3L1 was markedly increased in general ICU patients who developed AKI stage 2 or 3 within a 24-h period after their ICU admission. Urine CHI3L1 had a comparable diagnostic performance to TIMP-2•IGFBP7. Combining uCHI3L1 with the NephroCheck Risk® or IGFBP7 resulted in a less sensitive but a more specific test with the highest positive LRs (i.e., 1.69 to 2.75). However, LRs of this magnitude will generate a little effect on post-test disease probability in clinical practice [15].

This study and previous studies on uCHI3L1 have demonstrated its use as a biomarker for diagnosis of AKI, and this with comparable performance to uNGAL and NephroCheck Risk® [8, 9, 17–19]. CHI3L1 may also provide mechanistic insights in injury and repair mechanisms in the kidney. Kidney stress or damage will promote that macrophages in the kidney secrete CHI3L1 [17]. Biological effects of CHI3L1 include control of cell death, inflammation, and remodeling in renal epithelial cells and macrophages [20–22].

Notably, the diagnostic performance for AKI stage 2 or 3 within a 24-h period was markedly lower when compared to earlier validation studies for uCHI3L1 and NephroCheck Risk® [7, 8]. Indeed, we showed in a single-center general ICU cohort that for diagnosing AKI stage 2 or greater, uCHI3L1 had an AUC-ROC of 0.784 at 12 h and 0.721 at 24 h [8]. In the Sapphire study, the NephroCheck Risk® which combines the two biomarker urinary proteins TIMP-2 and IGFBP7 had an AUC-ROC of 0.80 at 12 h [5]. How should the current FINNAKI study data be interpreted? First, as AKI is a syndrome with marked heterogeneity, it is very likely that unmeasured confounders play a role in differences in the diagnostic performance of kidney biomarkers. While all 3 studies included patients from ICUs from developed countries in an apparently similar setting and with comparable age, comorbidities, and risk factors, there may have been differences in the baseline characteristics of patients. This hypothesis can be illustrated by the differences in the occurrence of AKI stage 2 or greater at 24 h. In the uCHI3L1 validation study, we found that 5% of patients developed AKI stage 2 or greater at 24 h compared to 7.4% in this study and to 14% within 12 h in the Sapphire study. A comparative overview of the baseline characteristics of these three studies (FINNAKI-CHI3L1 versus BAKI-I and Sapphire) is provided in Table 4. Strikingly, age, diabetes, baseline Cr, severity of illness, and type of ICU patients are different between studies. Apparently, a marked proportion of patients already have developed AKI stage 2 or greater when admitted to the ICU. Thus, potential interesting endpoints for future biomarker research would be the non-resolution of AKI. Second, the timing of diagnosis may explain the differences in AUC. Here, we measured AKI occurrence within a 24-h period after admission, a period during which ICU patients typically experience a complicated and eventful clinical course. Several new events may occur during this period resulting in injury or stress to the kidneys. A biomarker reading before these events occur will inevitably underestimate or miss this. Others have also reported lower AUCs for the NephroCheck Risk® when a longer detection period was used [23–25]. Third, the risk of progression to AKI stage 2 from baseline no AKI may be lower compared to baseline AKI stage 1. Therefore, a large proportion of no AKI patients at the time of inclusion may lower the diagnostic performance compared to studies in which a higher number of patients already had AKI stage 1 at inclusion. In our study, the majority of patients had no AKI (96.2%) at the time of inclusion. In Sapphire and BAKI-I, the number of patients who already had AKI stage 1 at the time of inclusion was not reported. Fourth, AKI is a syndrome with heterogeneous baseline characteristics. However, we consider our study population and findings representative to usual care in developed countries given the nationwide multicenter design, the internationally high standard of intensive care in Finland, consecutive inclusion of all patients with a deferred consent policy, a low rate of patients excluded without informed consent, and the exclusion of patients with AKI already at ICU admission from this analysis [26].

**Table 4:** Comparison of three AKI biomarker study characteristics

	Sapphire [5]	BAKI-I [8]	FINNAKI-CHI3L1 (present study)
*n*	728	181	660
Biomarker studied	TIMP-2•IGFBP7	CHI3L1	CHI3L1 TIMP-2•IGFBP7
Centers	Multi	Single	Multi
Age (years)	64 (53, 73)	60 (51, 70)	64 (53, 73)
Male gender (%)	62	63	62
Baseline Cr (μmol/L)	80	58	82
Diabetes (%)	29	7.2	21.2
SAPS II	NA	NA	38 (29, 49)
APACHE III	69 (51, 90)	NA	NA
SOFA	NA	9 (7, 11)	7 (4,9)
Emergency department	NA	41.4%	41.2%
Medical ICU admission (%)	31.0	59.7	70.9
AKI stages 2–3 < 12 h (%)	14.0	3.3	NA
AKI stages 2–3 < 24 h (%)	NA	4.9	7.4
AUC-ROC for AKI stages 2–3 < 12 h	0.80	0.79	NA
AUC-ROC for AKI stages 2–3 < 24 h	NA	0.72	uCHI3L1, 0.67 NephroCheck, 0.67

### Strengths and limitations

Our study has several strengths such as the multicenter study design with a diagnosis of AKI according to the full KDIGO definition and staging system in a large cohort of ICU patients. As such, this is the first external validation study of uCHI3L1 in general ICU patients compared with the NephroCheck Risk®, the current best-performing AKI biomarker for early diagnosis and kidney stress. Finally, we also assessed the diagnostic performance of uCHI3L1 in combination with the NephroCheck Risk® test and its individual elements. Therefore, we believe the results of this study are generalizable to patients admitted to ICUs in developed countries in similar settings.

Our study has also some limitations to be considered. First, we were able to include only 660 patients from the full cohort (*n* = 2901), because only a proportion of the full FINNAKI multicenter cohort had centrifuged frozen urine samples available. However, we consider possible bias as limited since these 660 patients represented the last consecutively admitted patients of the study. Second, plasma Cr was not sampled exactly at 24 h, but rather within a 12- to 25-h time frame. Third, although unlikely, we cannot exclude that pre-analytical issues may have resulted in false-negative biomarker measurements. For instance, despite deep-frozen storage at − 80 °C, the 7-year time delay between sample collection and analysis may have caused some protein degradation resulting in decreased detection of biomarkers. Fourth, patients with AKI stage 2 or greater were more severely ill, had higher baseline Cr, and more patients had pre-ICU risk factors. CHI3L1, TIMP-2, and IGFBP7 are mediators that are also elevated in the plasma in acute and chronic diseases [27–34]. Johnson and Zager demonstrated that TIMP-2 and IGFBP7 are filtered by the glomeruli [35]. Given the low molecular weight of 39–40 kDa, also CHI3L1 may be filtered by the glomeruli. In summary, we cannot exclude that systemic TIMP-2, IGFBP7, and CHI3L1 entered the urine by glomerular filtration and so impacted on the diagnostic performance. Fifth, serial measurement of biomarkers could have provided deeper insights into the biomarker signals.

## Conclusions

We found that uCHI3L1 and NephroCheck Risk® had a comparable diagnostic performance for diagnosis of AKI stage 2 or greater within a 24-h period in this multicenter FINNAKI cohort. In contrast to initial discovery and validation studies, the diagnostic performance was poor. Possible explanations for this observation were differences in patient populations, proportion of emergency admissions, proportion of functional AKI, rate of developing AKI, and observation periods for diagnosis of AKI. Our findings warrant additional multicenter validation studies for these biomarkers using consecutive critically ill patients and a scrutinized evaluation of additional clinical value before their wider implementation in clinical practice.

## Supplementary information

**Additional file 1: Table S1.** Diagnostic performance of biomarkers and biomarker combinations for any stage of AKI defined by KDIGO occurring at 24 hours (*n*= 120, 18.2%). **Table S2.** Diagnostic performance of biomarkers and biomarker combinations for any stage of AKI defined by KDIGO occurring at 48 hours (*n*=88, 13.3%). **Table S3.** Diagnostic performance of biomarkers and biomarker combinations for stage 2 or 3 AKI defined by KDIGO occurring at 48 hours (*n*=45, 6.8%).

## Data Availability

The dataset supporting the conclusions of this article is available from the corresponding author on reasonable request.

## Abbreviations

AKI: Acute kidney injury
ACCP/SCCM: American College Chest Physicians/Society of Critical Care Medicine
ADQI: Acute Dialysis Quality Initiative
AUC-ROC: Area under the curve of the receiver operating characteristic curve
BMI: Body mass index
CKD: Chronic kidney disease
Cr: Plasma creatinine concentration
ELISA: Enzyme-linked immunosorbent assay
FINNAKI: Finnish Acute Kidney Injury
GFR: Glomerular filtration rate
ICU: Intensive care unit
IGFBP7: Insulin-like growth factor-binding protein 7
IQR: Interquartile range
KDIGO: Kidney Disease: Improving Global Outcomes
LR: Likelihood ratio
MDRD: Modification of Diet in Renal Disease
NA: Not available
RRT: Renal replacement therapy
SAPS II: Simplified Acute Physiology Score II score
SOFA: Sequential Organ Failure Assessment score
TIMP-2: Tissue inhibitor of metalloProteinases-2
uCHI3L1: Urinary chitinase 3-like protein 1
uNGAL: Urinary neutrophil gelatinase-associated lipocalin
UO: Urine output
