# Supplementary material for: Urinary cell cycle arrest biomarkers and chitinase 3-like protein 1 (CHI3L1) to detect acute kidney injury in the critically ill: a post hoc laboratory analysis on the FINNAKI cohort
Source: Crit Care. 2020 Apr 10;24:144. doi: 10.1186/s13054-020-02867-w (PMC7149885; doi:10.1186/s13054-020-02867-w)
Supplement: Supplementary file 1 — Additional file 1: Table S1. Diagnostic performance of biomarkers and biomarker combinations for any stage of AKI defined by KDIGO occurring at 24 hours (n= 120, 18.2%). Table S2. Diagnostic performance of biomarkers and biomarker combinations for any stage of AKI defined by KDIGO occurring at 48 hours (n=88, 13.3%). Table S3. Diagnostic performance of biomarkers and biomarker combinations for stage 2 or 3 AKI defined by KDIGO occurring at 48 hours (n=45, 6.8%). [file 13054_2020_2867_MOESM1_ESM.pdf]

Additional file table 1. Diagnostic performance of biomarkers and biomarker combinations for any stage of AKI defined by KDIGO occurring at 24 hours (n= 120, 18.2%).

Biomarker	Area under the curve	Cut off value	Sensitivity	Specificity	Positive LR	Negative LR
uCHI3L1 (ng/mL)	0.651 (0.593-0.708)	>3.1	48.3% (39.1-57.6)	81.4% (77.9-84.7)	2.61 (2.02-3.37)	0.63 (0.53-0.76)
NephroCheck Risk® $(ng/mL)^2/1000$	0.681 (0.626-0.737)	>0.3	68.3% (59.2-76.5)	59.4% (55.2-63.6)	1.68 (1.44-1.98)	0.53 (0.41-0.70)
NephroCheck Risk® $(ng/mL)^2/1000$		>2.0	28.3% (20.5-37.3)	89.8% (86.9-92.2)	2.78 (1.90-4.06)	0.80 (0.71-0.90)
uCHI3L1•TIMP-2 $(ng/mL)^2$	0.692 (0.639-0.745)	>3.73	64.2% (54.9-72.7)	65.7% (61.6-69.7)	1.87 (1.57-2.24)	0.55 (0.43-0.70)
uCHI3L1•IGFBP7 $(ng/mL)^2$	0.674 (0.618-0.729)	>507.1	45.0% (35.9-54.3)	84.1% (80.7-87.1)	2.83 (2.14-3.73)	0.65 (0.55-0.77)
uCH3L1• NephroCheck Risk® $(ng/mL)^3/1000$	0.688 (0.634-0.743)	>1.28	51.7% (42.4-50.9)	78.0% (74.2-81.4)	2.34 (1.85-2.96)	0.62 (0.51-0.75)

95% confidence intervals in parenthesis.

LR; likelihood ratio

Additional file table 2. Diagnostic performance of biomarkers and biomarker combinations for any stage of AKI defined by KDIGO occurring at 48 hours (n=88, 13.3%).

Biomarker	Area under the curve	Cut off value	Sensitivity	Specificity	Positive LR	Negative LR
uCHI3L1 (ng/mL)	0.623 (0.555-0.691)	>2.1	54.5% (43.6-65.2)	73.8% (70.0-77.3)	2.08 (1.64-2.63)	0.62% (0.49-0.78)
NephroCheck Risk® $(ng/mL)^2/1000$	0.662 (0.599-0.726)	>0.3	65.9% (55.0-75.7)	57.5% (53.3-61.6)	1.55 (1.30-1.85)	0.59 (0.44-0.80)
NephroCheck Risk® $(ng/mL)^2/1000$		>2.0	28.4% (19.3-39.0)	88.8% (85.9-91.2)	2.54 (1.69-3.80)	0.81 (0.70-0.92)
uCHI3L1•TIMP-2 $(ng/mL)^2$	0.668 (0.606-0.730)	>10.3	52.3% (41.4-63.0)	74.3% (70.5-77.8)	2.03 (1.59-2.59)	0.91 (0.87-0.92)
uCHI3L1•IGFBP7 $(ng/mL)^2$	0.642 (0.578-0.706)	>325.4	45.5% (34.8-56.4)	79.5% (76.0-82.8)	2.22 (1.68-2.94)	0.69 (0.56-0.83)
uCH3L1• NephroCheck Risk® $(ng/mL)^3/1000$	0.659 (0.596-0.723)	>1.1	51.1% (40.2-61.9)	75.0% (71.2-78.5)	2.04 (1.60-2.62)	0.65 (0.52-0.81)

95% confidence intervals in parenthesis.

LR; likelihood ratio

Additional file table 3. Diagnostic performance of biomarkers and biomarker combinations for stage 2 or 3 AKI defined by KDIGO occurring at 48 hours (n=45, 6.8%).

Biomarker	Area under the curve	Cut off value	Sensitivity	Specificity	Positive LR	Negative LR
uCHI3L1 (ng/mL)	0.645 (0.554-0.736)	>2.1	60.0% (44.3-74.3)	72.7% (70.0-76.2)	2.20 (1.67-2.88)	0.55 (0.38-0.79)
NephroCheck Risk® (ng/mL)^2/1000	0.668 (0.581-0.755)	>0.3	68.9% (53.4-81.8)	56.1% (52.1-0.60)	1.57 (1.26-1.95)	0.55 (0.36-0.86)
NephroCheck Risk® (ng/mL)^2/1000		>2.0	28.8% (16.4-44.3)	87.6% (84.8-90.1)	2.34 (1.41-3.87)	0.94 (0.92-0.96)
uCHI3L1•TIMP-2 (ng/mL)^2	0.695 (0.618-0.771)	>4.5	66.7% (51.0-80.0)	65.0% (61.1-68.8)	1.91 (1.51-2.41)	0.51 (0.34-0.78)
uCHI3L1•IGFBP7 (ng/mL)^2	0.658 (0.576-0.741)	>507.1	46.7% (31.7-62.1)	80.7% (77.3-83.7)	2.41 (1.70-3.43)	0.66 (0.50-0.87)
uCH3L1• NephroCheck Risk® (ng/mL)^3/1000	0.675 (0.591-0.760)	>0.11	77.8% (62.9-88.8)	52.7% (48.7-56.7)	1.64 (1.38-1.96)	0.42 (0.24-0.73)

95% confidence intervals in parenthesis.

LR; likelihood ratio
